# Can High-Dimensional Questionnaires Resolve the Ipsativity Issue of Forced-Choice Response Formats?

**DOI:** 10.1177/0013164420934861

**Published:** 2020-07-24

**Authors:** Niklas Schulte, Heinz Holling, Paul-Christian Bürkner

**Affiliations:** 1 University of Münster, Germany; 2 Aalto University, Espoo, Finland

**Keywords:** forced-choice format, Thurstonian IRT model, ipsative data, multidimensional IRT

## Abstract

Forced-choice questionnaires can prevent faking and other response biases typically associated with rating scales. However, the derived trait scores are often unreliable and ipsative, making interindividual comparisons in high-stakes situations impossible. Several studies suggest that these problems vanish if the number of measured traits is high. To determine the necessary number of traits under varying sample sizes, factor loadings, and intertrait correlations, simulations were performed for the two most widely used scoring methods, namely the classical (ipsative) approach and Thurstonian item response theory (IRT) models. Results demonstrate that while especially Thurstonian IRT models perform well under ideal conditions, both methods yield insufficient reliabilities in most conditions resembling applied contexts. Moreover, not only the classical estimates but also the Thurstonian IRT estimates for questionnaires with equally keyed items remain (partially) ipsative, even when the number of traits is very high (i.e., 30). This result not only questions earlier assumptions regarding the use of classical scores in high-dimensional questionnaires, but it also raises doubts about many validation studies on Thurstonian IRT models because correlations of (partially) ipsative scores with external criteria cannot be interpreted in a usual way.

Psychometric assessments largely depend on self-reports based on Likert-type scales, but these rating scales are susceptible to a number of biases such as acquiescence, extremity/midpoint bias, leniency/severity tendencies, and faking attempts (Paulhus & Vazire, 2007; Wetzel, Böhnke, & Brown, 2016), reference group effects (Credé et al., 2010) as well as idiosyncratic interpretations of anchor labels (Wetzel & Greiff, 2018). Each of these effects can compromise the validity of the derived factor scores. To overcome these issues, forced-choice (FC) scales have been proposed, where respondents have to choose between (or rank) two or more equally attractive items. Omitting ratings by design, this technique eliminates all rating scale-related biases (acquiescence, extremity, leniency, etc.). Most important, according to a meta-analysis by Cao and Drasgow (2019), FC tests are an effective approach to drastically reduce faking and – if tests are constructed and scored appropriately – even have the potential to completely prevent score inflation in a socially desirable direction.

Researchers have been employing FC questionnaires not only in high-stakes situations like personnel selection (Christiansen et al., 2005; Stewart et al., 2010) and performance appraisals (Brown et al., 2017) but also for constructs in which highly socially desirable response behavior is to be expected such as value measurement (van Eijnatten et al., 2015; Merk et al., 2017) and dark triad measurement (Paulhus & Jones, 2014; Young, 2018). Other areas of application are market research (Parvin & Wang, 2014) and comparative cultural studies where response biases vary between cultures (He et al., 2014; T. Johnson et al., 2005).

The main challenge associated with FC scales is the amount and nature of information included in the answers. Traditional scoring approaches yield ipsative scores, that is, the scores of each individual on different dimensions sum up to the same total, making comparisons between individuals impossible (Hicks, 1970). The most widely used method that has been designed to solve this issue is the Thurstonian item response theory (T-IRT) model (Brown & Maydeu-Olivares, 2011); as this model offers the promise of faking-resistant normative scores in high-stakes situations, it has been the subject of considerable research. Many for-profit test developers have adjusted their instruments accordingly and a substantial part of the literature on T-IRT has been (co)authored by for-profit test developers (e.g., Anguiano-Carrasco et al., 2015; Brown & Bartram, 2013; Brown et al., 2017; Joubert et al., 2015; Lewis, 2015; Lin & Brown, 2017; Walton et al., 2019; Watrin et al., 2019), demonstrating the practical relevance of the model.

Yet a recent simulation suggests that simply changing the parameter estimation method does not lead to sufficiently reliable estimates for many FC questionnaires (Bürkner et al., 2019). When the simulated questionnaires measured five or fewer traits, the model failed to reach a satisfactory level of measurement precision in all practically relevant conditions. They performed especially poorly when the factor loadings of all items had the same sign within one block, that is, were equally keyed. Item keying refers to the sign of the factor loading (not to their absolute value). Its influence on the reliability of factor scores was already described in the first publication on T-IRT models (Brown & Maydeu-Olivares, 2011). Equally keyed items are a necessary – although not sufficient – condition for maintaining the resistance to faking in practice. Given a pair of two mixed keyed items, the positively keyed item will typically cover the desired end of the trait continuum, while the negatively keyed item will represent the undesired end of the corresponding trait. Therefore, in a high-stakes situation, many respondents will engage in socially desirable responding and choose the more desirable item (the same argument applies if negatively keyed items cover the desirable end of the continuum).

In contrast to T-IRT models that measure few traits, T-IRT models have performed well when measuring 30 traits even with only equally keyed items (Bürkner et al., 2019). Even though this trait number represents the most prominent FC personnel selection test, namely the Occupational Personality Questionnaire (OPQ; Brown & Bartram, 2013), most test constructors seek to develop tests with fewer traits. Indeed, inductive methods of test development will rarely suggest such a high number of dimensions, and, in theory-driven approaches, they violate the principle of parsimony. Therefore, most of the popular personality models (e.g., Big Five, Dark Triad) consist of significantly fewer dimensions. This is also reflected in the tests evaluated with T-IRT so far, which typically measure between three and 15 dimensions (e.g., Anguiano-Carrasco et al., 2015; Guenole et al., 2018; Lewis, 2015; Merk et al., 2017; Watrin et al., 2019). The practical utility of the T-IRT method therefore largely depends on its ability to derive precise reliable person parameter estimates from questionnaires with only equally keyed items and fewer than 30 traits. Until now, it has been unknown how T-IRT models perform when measuring between five and 30 traits. The present simulation study seeks to establish the minimal trait number that is required to obtain person parameter estimates from FC questionnaires analyzed with T-IRT that show a high (squared) correlation with true trait scores (i.e., a high reliability) and low absolute estimation errors (i.e., a low root mean square error [RMSE]).

Before elaborated analysis strategies such as T-IRT became available recently, FC tests were scored with very simple methods that basically rely on assigning points to the latent dimension each time an item of this dimension is picked or is highly ranked (Hontangas et al., 2015). These approaches result in ipsative person parameters, such that the score of an individual on one trait is dependent on that individual’s score on the other measured traits. Such scores are said to be inappropriate for interindividual comparisons (Cattell, 1944; Hicks, 1970) and are associated with a number of psychometric issues (Baron, 1996). Remarkably, advocates of these scoring procedures have demonstrated that classical ipsative scores can be reliable and highly correlated to normative scores if a large number of traits (such as 30) is measured (Baron, 1996; Bartram, 1996; Saville & Willson, 1991). This raises the question of how much better T-IRT estimates are compared with traditional scoring methods. On one hand, we can only identify critical issues such as low reliability and biased intertrait correlation estimates for T-IRT models in conditions under which traditional scoring methods fail (i.e., when measuring up to five traits; Bartram, 1996), whereas, on the other hand, T-IRT seems to yield good results under conditions in which traditional scoring procedures yield sensible results, too. These observations suggest that some designs are inherently less informative than others. It is, however, unknown whether T-IRT really extends the range of questionnaires that can be soundly analyzed compared with other longstanding and much simpler approaches. While the incorporation of factor loadings and trait intercorrelations by T-IRT models can be expected to offer some improvement in measurement precision, the size – and therefore the practical relevance of this improvement – is unknown to date.

Furthermore, earlier simulations were designed to establish the measurement precision (i.e., reliability and RMSE) of FC questionnaires under optimal conditions. Therefore, the present study seeks to evaluate the measurement precision under less optimal circumstances such as smaller sample sizes and lower (and perhaps more realistic) factor loadings. With regard to the latter, the favorable judgments on the quality of T-IRT estimates from high-dimensional questionnaires, for instance, are based on simulations with (what we now think are) unusually high factor loadings between .65 and .95 (Bürkner et al., 2019). In the present study, we will additionally use factor loadings derived from the NEO-PI-R (Costa & McCrae, 1992; Ostendorf & Angleitner, 2004) – the most widely used high-dimensional personality questionnaire – leading to more realistic loading patterns in the simulated questionnaires.

The present study was designed to determine which FC designs that have the potential to be faking resistant (through the use of only equally keyed factor items) can under realistic conditions (i.e., with varying sample sizes and typical factor loadings) yield acceptable psychometric properties. Further, the comparison of classical and T-IRT scoring will demonstrate the influence of the estimation procedure.

## The Thurstonian IRT Model

The Thurstonian IRT model has been described in detail elsewhere (e.g., Brown & Maydeu-Olivares, 2011, 2012; Bürkner et al., 2019), so we keep its introduction brief. Independent of the exact format of the forced-choice questionnaire, that is, whether the responses imply a full or partial ranking of all alternatives in a given block, responses can be represented by a set of pairwise binary choices (Brown & Maydeu-Olivares, 2011). We write $y_{\mathrm{pik}}$ to indicate the binary choice of person p between two items i and k . We set $y_{\mathrm{pik}}=1$ if the person’s response indicates that they prefer item i over item k and set $y_{\mathrm{pik}}=0$ otherwise. Under a Thurstonian model, we assume that each item i evokes a latent utility $t_{\mathrm{pi}}$ which describes the psychological value or desirableness of item i for person p (Maydeu-Olivares & Böckenholt, 2005; Thurstone, 1927). If we further assume a unidimensional factor model for $t_{\mathrm{pi}}$ based on some univariate person trait $\eta_{a}$ (Brown & Maydeu-Olivares, 2011; Bürkner et al., 2019), we can write

(1) $$t_{\mathrm{pi}}=\mu_{i}+\lambda_{i}\eta_{\mathrm{ap}}+\varepsilon_{\mathrm{pi}},$$

where $\mu_{i}$ is the mean of the latent utility across persons, $\lambda_{i}$ is the factor loading on trait $\eta_{a}$ , and $\varepsilon_{\mathrm{pi}}$ is an independently normally distributed error term with variance $\psi_{i}^{2}$ . In Thurstonian IRT models, only the differences between the utilities $t_{i}$ and $t_{k}$ are of interest, and so we define $y_{\mathrm{pik}}^{*}=t_{\mathrm{pi}}-t_{\mathrm{pk}}$ as the latent preference of person p for item i over item k . This implies that $y_{\mathrm{pik}}=1$ if $y_{\mathrm{pik}}^{*}\geq 0$ and $y_{\mathrm{pik}}=0$ otherwise. Supposing that item i loads on trait $\eta_{a}$ and item k loads on trait $\eta_{b}$ , we can write $y_{\mathrm{pik}}^{*}$ as

(2) $$y_{\mathrm{pik}}^{*}=\mu_{i}+\lambda_{i}\eta_{\mathrm{ap}}+\varepsilon_{\mathrm{pi}}-\mu_{k}-\lambda_{k}\eta_{\mathrm{bp}}-\varepsilon_{\mathrm{pk}}.$$

Following Brown and Maydeu-Olivares (2011), we simplify the mean structure and set $-\gamma_{\mathrm{ik}}=\mu_{i}-\mu_{k}$ so that each pair of compared items effectively has its own mean parameter. Under the additional assumption that all traits η are multivariate normal with correlation matrix Φ as well as, for identification, means of 0 and variances of 1 , we can formulate the probability that $y_{\mathrm{pik}}=1$ as

(3) $$P(y_{\mathrm{pik}}=1)=\Phi \left(\frac{-\gamma_{\mathrm{ik}}+\lambda_{i}\eta_{\mathrm{ap}}-\lambda_{k}\eta_{\mathrm{bp}}}{\sqrt{\psi_{i}^{2}+\psi_{k}^{2}}}\right).$$

If we aim to use Equation (3) as the pointwise likelihood outside of a structural equation framework, additional item-specific terms have to be incorporated to account for the residual dependency between different comparisons involving the same item (see Bürkner et al., 2019, for details).

### Evidence on Model Properties From Validations With Real-World Data

The promising prospect of faking-resistant normative scores has impelled many test developers to switch over from classical (ipsative) scoring procedures to T-IRT or to develop new FC-based tests scored with T-IRT (e.g., Anguiano-Carrasco et al., 2015; Brown & Bartram, 2013; Guenole et al., 2018; Lewis, 2015; Merk et al., 2017; Watrin et al., 2019). However, in order to really judge the model’s validity and utility, scholars have engaged in a number of validation studies. These can be roughly divided into the following categories: attempts to evaluate the appropriateness of the theoretical model, which describes how respondents form their answers; studies that compare the model with traditional scoring methods; and investigations that examine the question of whether T-IRT FC scales yield more valid parameter estimates than rating scales.

With respect to the theoretical assumptions on the response process, respondents have indeed reported in a think-aloud task that they make pairwise comparisons between all items of a block (Sass et al., 2018). In that study, where blocks of three items were used, 76% of participants reported to have no difficulty keeping in mind the information related to all statements in order to appraise the relative utility of all items. Thus, the *Law of Comparative Judgement* (Thurstone, 1927) seems to be a reasonable explanation for the response process in most of the respondents. However, it is likely that larger blocks will lead to more severe violations of model assumptions, as cognitive load increases with higher numbers of necessary comparisons. When asked directly, participants have stated that it is harder to express accurately their actual attitude toward the items in FC compared with rating formats (Watrin et al., 2019).

Regarding studies that have compared the T-IRT model with traditional scoring methods, results are mixed. One study found more consistent correlations with external criteria for T-IRT scores (Anguiano-Carrasco et al., 2015), two found very similar results for both methods (Lee et al., 2018; Wetzel, Roberts, et al., 2016, with an advantage for T-IRT on one subscale) and one study observed similar convergent but problematic discriminant validities for T-IRT scores (Walton et al., 2019). Counterintuitively, in one study T-IRT scores did not predict job performance (mean ρ=.00 ) while the classical (ipsative) scores for the same questionnaire did (mean ρ=.38 ; Fisher et al., 2019). Together, there is no reason to expect improved validity for T-IRT per se when compared with classical scoring approaches based on the current data.

The third line of validation studies compares FC T-IRT scales with rating scales. These studies are much more challenging to conduct and interpret as the results of a specific validation study depend on and are limited to the specifications of the concrete test at hand (keyed direction of items, number of traits, trait intercorrelations, etc.) and the situation of the study (e.g., high-stakes vs. low-stakes). Unlike when comparing several scoring methods for the same questionnaire, here the source of the data (FC vs. rating) differs, and different sources might be differentially affected by the variation in the questionnaires and the situational characteristics. Nonetheless, previous validation efforts have provided some insights. FC T-IRT scores correlate substantially with corresponding rating scales (on average .76 in Guenole et al., 2018, .79 in Lee et al., 2018, and .62 in Watrin et al., 2019). The associations are high, but together with differential loading patterns (Guenole et al., 2018), these results suggest at least slightly variant trait meanings. In another study, T-IRT estimates resulted in more stable personality profile solutions between honest and faking conditions, and ratings scales turned out to be more fakeable than FC T-IRT estimates for participants with an extreme level of faking tendency (Lee et al., 2019). Comparing the validity of FC T-IRT and rating scales, three studies report equal or somewhat lower validities for FC T-IRT (Anguiano-Carrasco et al., 2015; Brown & Maydeu-Olivares, 2013; Lee et al., 2018), and one study reports comparable divergent validities but slightly better predictions of school grades as a criterion (Watrin et al., 2019). One limitation of most of these studies is the use of validation scales with a rating format. Such scales share common-method variance with the rating version of the evaluated questionnaire, and the horse-race approach is therefore biased in favor of the rating scale. Only one study incorporated both FC and rating scale versions of the validated (Big Five) and validating (HEXACO) questionnaires as well as other-ratings (Wetzel & Frick, 2019). In this study, some intertrait correlations of the FC questionnaire differed drastically from those of the rating scale version of the same questionnaire and from meta-analytic estimates for Big Five intercorrelations, suggesting potential issues in the estimation of intertrait correlations. Convergent validities with another Big Five questionnaire and other-ratings as well as cross-response format comparisons in Big Five and HEXACO questionnaires did not result in consistent evidence in favor of T-IRT FC. In this study, there was no relevant difference between T-IRT FC and rating scales in the prediction of a variety of criteria. However, all of the aforementioned studies were conducted under low-stakes situations in which respondents are hardly expected to fake, such that in these situations FC questionnaires will not benefit from their potential resistance to faking. Indeed, in the only study conducted in a medium-stakes situation (360° feedback) using an FC scale as a criterion, validity estimates of FC T-IRT scales were superior compared with rating scales (Brown et al., 2017). Yet in that study, rating scales analyzed with a special bias factor reached convergent validity levels that were equal to those of T-IRT estimates.

In sum, the literature on the validity of T-IRT estimates does not draw a clear picture in favor of FC-based T-IRT estimates compared with rating scales or traditionally scored FC scales. Yet one viable way to understand which aspects of the questionnaire design might have contributed to these mixed findings and to identify aspects that can promote precise parameter estimates is by performing simulation studies. Next, we will summarize evidence from prior simulations.

### Evidence From Earlier Simulations

Two simulations studies investigated how well item parameters and latent trait scores can be recovered (Brown & Maydeu-Olivares, 2011; Bürkner et al., 2019; but see also, Xiao et al., 2017).

The first simulations were performed for the initial publication of the T-IRT model (Brown & Maydeu-Olivares, 2011). This study showed that the factors’ test length (12 or 24 items per trait), correlation among traits (0, .5, −.5), and block size (two, three, or four items per trait were simulated) all affect the accuracy of item and person parameters. However, by far the most important aspect is the keyed direction of items (only equally keyed vs. mixed keyed). More specifically, T-IRT performs well in most conditions where mixed keyed items are used. In contrast, the model’s ability to reliably estimate person parameters with only positively keyed items was brought into question as the squared correlation between true and estimated trait scores was unsatisfactorily low. Equally keyed items seem to recover differences between traits (i.e., their *relative* position) very well but contribute little to the estimation of their sums, and, thus, are less useful for recovering the trait’s *absolute* location (Brown & Maydeu-Olivares, 2011). This issue is further exacerbated when both measured traits are positively correlated (see Equations 21 and 22 as well as Table 3 in Brown & Maydeu-Olivares, 2011).

However, the ability of FC tests to rely on equally keyed items is very important for their faking resistance. It is only when respondents have to choose between equally desired options that the rationale behind FC items can prevent socially desirable responding. This prerequisite is violated on a regular basis if one item with a positive and one item with a negative factor loading are compared within the same block. Without loss of generality, we assume that higher trait values are more desirable for the traits we are concerned with (the argument works regardless of that assumption). In this case, positively keyed items will represent the desired end and negatively keyed items the undesired end of one trait continuum, making comparisons with unequally keyed items prone to socially desirable responding. However, FC tests’ anticipated faking resistance was the primary reason for introducing FC techniques in contexts like personnel selection or performance appraisals and accepting some possibility of faking in exchange for reliable estimates might be misleading. For example, the reliability level that simulation studies suggest for equally keyed items is based on a generative model assuming that respondents will choose the item which describes them best. If respondents can identify an “optimal”– that is, social desirable item – many will choose (or highly rank) this item, and the respective block will contribute less information to the parameter estimation (Wang et al., 2017). Therefore, employing mixed keyed items in high-stakes situations may result in much lower reliabilities than simulations suggest. It should be mentioned that inverting individual traits (e.g., neuroticism instead of emotional stability) or mixing desired and undesired traits (e.g., Big Five and Dark Triad) only relocates the problem. This is because FC questionnaires need both equally and unequally keyed comparisons for each trait to estimate factor scores precisely (see the section on scoring procedures). For a detailed discussion of problems arising from mixed keyed items in FC questionnaires, see Bürkner et al. (2019).

In a second simulation study (Bürkner et al., 2019), neither frequentist estimation via Mplus (Muthén & Muthén, 2015) and lavaan (Rosseel, 2012) nor a Bayesian estimation via Stan (Carpenter et al., 2017) yielded sufficiently accurate trait scores for most conditions in which equally keyed items were used. Intertrait correlation estimates showed a considerable bias in these conditions, suggesting that person parameters remained partially ipsative. Individual standard errors were more than twice as high for very high and very low scores than for average trait scores. Thus, in applied contexts, extremely (un)qualified applicants, employees, and so on, cannot easily be separated from average test-takers which is the very reason for administering such questionnaires. Nevertheless, this study also gave hints on how to improve measurement precision even when only equally keyed items are used: In a condition with 30 traits and a realistic intertrait correlation matrix (i.e., heterogeneous correlations taken from a real-world questionnaire), the model yielded a very high reliability, and intertrait correlations were estimated without relevant bias.

The results of the latter study raise three different questions. First, the excellent measurement precision in the condition with 30 traits suggests that fewer than 30 traits would likewise result in adequate reliability levels. Though, until now there has been no evidence on model properties in the range of five and 30 traits, because earlier simulations only investigated up to five traits and the isolated case of 30 traits. Second, how much do simulation results depend on the high factor loadings used so far? These questions will be subject to the simulations presented. Third, some have claimed that traditional scoring methods can also yield non-ipsative scores if the number of measured traits is high (Bartram, 1996; Saville & Willson, 1991); we will discuss this fact in the following section.

### Classical Scoring Approaches for Forced-Choice Items

Until recently, FC questionnaires have been scored following a very simple procedure. For the RANK format for instance, in which the task is to rank all items of a block, the inverted rank order of the items is added to their respective scales. Given a block of three items, the scale of the most highly ranked item is assigned three points, the second two points and the least preferred item one point. Of course, all linear transformations of this scoring – such as {−1, 0, 1} – are permitted and have no influence on the diagnostic information the scores contain. Scorings for other tasks like MOLE (choose the MOst and LEast decriptive item) and PICK (pick one item) slightly differ but follow the same rationale, as they represent partial rankings (for an overview, see Hontangas et al., 2015). There is no consent in the literature on how to treat mixed keyed items in such scorings. With few exceptions (e.g., Lee et al., 2018), this question is largely ignored in most relevant articles.

Irrespective of that point, we can state for positively keyed items that this scoring procedure yields *ipsative* person parameter estimates (Saville & Willson, 1991). A measure is ipsative if all measured dimensions sum to the same total for each individual (Clemans, 1966). The units of ipsative scales are relative to other measurements on the same person (Cattell, 1944). Consequently, each individual’s score depends on their own score on all other dimensions and is not comparable with scores of other respondents. However, most measurements require *normative* person parameters. A measurement is normative if “subjects are placed in order relative to one another and assigned a standard score in terms of the population distribution” (Cattell, 1944, p. 293). For a discussion of statistical and psychometric problems of ipsative scales, see not only Hicks (1970), and Baron (1996) but also consider C. E. Johnson et al. (1988) as well as Meade (2004) for issues in personnel selection.

Besides all criticism, proponents of ipsative FC scales have argued that a sweeping rejection of classical scoring approaches is unreasonable. It seems that under certain circumstances, reliable and valid person parameters can be derived from ipsative questionnaires and that these scores can be interpreted like normative measures (Baron, 1996; Bartram, 1996; Saville & Willson, 1991). In one simulation, classically scored ipsative questionnaires correlated highly with true scores when measuring 32 traits and correlated more weakly but still on a high level when fewer traits were simulated (16 and seven traits respectively; Saville & Willson, 1991). Saville and Willson (1991) also report comparable validity estimates for the rating and FC version of a 30-trait real-world questionnaire (but, see Cornwell & Dunlap, 1994, for critical response). A second simulation confirmed that with 30 scales and low intertrait correlations, ipsative scores are both reliable and highly correlated with normative ones (Bartram, 1996). We assume the underlying reason for the positive effect of higher trait numbers to be the following: Imagine two questionnaires with ideal items (factor loadings of 1, all-zero intercorrelations), one with two and another with 30 traits being measured. Now, for example, a person has an extremely high score on Trait 1, a slightly lower but still high score on Trait 2, somewhat lower scores on the other traits. Further, for simplicity, suppose that the response model is fully deterministic. Under these assumptions and the *Law of Comparative Judgement* (Thurstone, 1927), the person will always reject the items of Trait 2 in the two-trait questionnaire resulting in a very low score on Trait 2. In the 30-trait questionnaire, the person does not have to reject all items of Trait 2, but can express this trait’s high value in the preference of this trait’s items in comparisons were items of Trait 1 are not involved, resulting in a more realistic estimate of Trait 2. More generally, the more characteristics are measured, the more nuances in characteristic differences can be expressed in the responses.

Taken together, we can state that T-IRT models only yield acceptable measurement precision under exactly those conditions under which the much simpler traditional scoring approaches also seem to work quite well. Because T-IRT models incorporate factor loadings and intertrait correlations into person parameter estimation, we expect T-IRT to yield at least equal measurement precision and, in all applied conditions (i.e., nonzero intercorrelations, factor loadings less than 1), superior measurement precision. Though, whether the increase in precision is substantial enough to justify the much more complicated estimation procedure is an unanswered question. Additionally, a direct comparison of both scoring formats is of interest because several validation studies compare both formats, and results do not necessarily indicate the superiority of T-IRT as compared with classical scoring approaches (Lee et al., 2018; Walton et al., 2019; Wetzel, Roberts, et al., 2016).

## Method

### Simulation Conditions

We simulated a maximum of 30 traits because this amount has been repeatedly discussed as a number at which even traditional scoring methods are free from most limitations of ipsative scores (Baron, 1996; Bartram, 1996; Saville & Willson, 1991). Additionally, the most prominent commercial FC questionnaire, the OPQ, measures – depending on the version – 30 or 32 traits (Brown & Bartram, 2011; Saville et al., 1992). Sample sizes in earlier simulations were comparably high (1,000 observations in Brown & Maydeu-Olivares, 2011, and 2,000 observations in Bürkner et al., 2019). Such high sample sizes optimize the estimation of model parameters, but in practice, samples of this size are not always available. In order to examine the model properties under conditions which are realistic for companies applying personnel selection tests or researchers conducting a laboratory study, the sample size was varied between N=100 and N=1000 (see below for details). Further, we aimed to assure a realistic level of factor loadings. Prior simulations draw loadings from a uniform distribution between .65 and .95 (Brown & Maydeu-Olivares, 2011) or between .65 and .95 and additionally between .3 and .7 for some conditions (Bürkner et al., 2019). However, in the one condition in which T-IRT models estimated person parameters precisely and resistantly against faking (i.e., with 30 traits and only equally keyed items within a block) only loadings between .65 and .95 were simulated. This is unsatisfactory, as factor loadings are often lower in practice. A well-known psychometric questionnaire measuring 30 traits is the NEO-PI-R (Costa & McCrae, 1992). We conducted a CFA with the norming sample of the German version (Ostendorf & Angleitner, 2004) and found factor loadings to be approximately normally distributed with M=0.5 and SD=0.16 . This empirical approach was used to simulate realistic factor loadings based on a real-world questionnaire. Additionally, we sampled factor loadings uniformly between .65 and .95 to maintain comparability with Brown and Maydeu-Olivares (2011) as well as Bürkner et al. (2019) and to see what effect higher factor loadings would have.

Together, 384 conditions were examined by crossing the following factors: (a) scoring procedure (classic vs. T-IRT); (b) number of traits *N_T_* from five to 30 in steps of 5; (c) sample size N with the levels 100, 300, 500, and 1,000; (d) keyed direction of items with either all positive factor loadings (referred to as equally keyed) or with one half of the factor loadings having positive and one half having negative factor loadings (termed *mixed* or *unequally keyed*); (e) factor loadings drawn from a uniform distribution between .65 and .95 or truncated normal distribution with M=0.5 and SD=0.16 within the limits of 0.1 and 0.9; (f) intertrait correlations which were either all set to 0 or taken from the German version of the NEO-PI-R (Ostendorf & Angleitner, 2004) to represent a set of realistic intercorrelations. Because the NEO-PI-R measures six facets of five dimensions, the subset of intercorrelations was changed six times within each condition. We balanced the number of facets per dimension within each matrix. Each correlation matrix was used for two replications leading to 6×2=12 replications per condition. The exact mechanism for matrix extraction is documented in the scripts on OSF (Open Science Framework; https://osf.io/t6krs/). The number of comparisons between traits was not balanced, which means that not every trait was compared equally frequently with every other trait. In the case of high-dimensional questionnaires, such a balancing leads to a questionnaire length that in practice can hardly be implemented.

The following simulation factors were held constant: the number of items per block was 3 (i.e., triplets), as it is the most widely used block size. Besides, with larger block sizes, the number of paired comparisons necessary to rank all items of a block drastically increases and the *Law of Comparative Judgement* (Thurstone, 1927) might no longer be a reasonable model for participants’ response process. We use the RANK format, as it contains the maximum amount of information compared with all other response formats and its superiority has been demonstrated expectedly in earlier simulations (Hontangas et al., 2015). In the case of triplets, which we consider here, RANK and MOLE formats are equivalent, because the option that is chosen as neither the most nor the least descriptive is always in the second ranking position. Further, we simulated nine blocks per trait. Despite the fact that higher block numbers can slightly improve estimate precision (Bürkner et al., 2019), they are unrealistic for the range of trait numbers considered here. The error variances ψ were computed as $\psi =1-\lambda^{2}$ as we simulated standardized factor loadings. The γ values (i.e., the models’ intercepts) were sampled uniformly between −1 and 1.

Most of the conditions described here were preregistered (see https://osf.io/t6krs/). The condition of high factor loadings [0.65,0.95] was added in the light of the other results to explain why measurement precision falls off notably compared with the results in Bürkner et al. (2019) under the preregistered conditions. The sample size was initially fixed to N=1000 but during the research process, we gained access to high-performance computing capacities and expanded our design. In fact, to readers interested in applying T-IRT models, we want to point out that most of the models specified here take several days to be estimated with today’s computer performance when estimated with full Bayesian estimation methods. Frequentist methods should be faster but are often accompanied by convergence problems (Bürkner et al., 2019).

### Scoring Procedures

The traditional scores were obtained as follows. Analogous to T-IRT procedures, ratings were first transformed into paired comparisons (Brown & Maydeu-Olivares, 2011; Maydeu-Olivares & Böckenholt, 2005). Table 1 shows how pairs are scored depending on the sign of the factor loading and the preferred item within the paired comparison. For instance, if both items are positively keyed (first row) and the first item is preferred over the second then a 1 is assigned to the trait score of the first item and a 0 to the trait score of the second item’s latent dimension. This procedure is repeated for each binary outcome of the paired comparisons. Thus, the trait score is a sum of the assigned values.

**Table 1.** Classical Scoring Scheme.

Sign of factor loading		Value added to trait if $y_{\mathrm{ik}}=1$		Value added to trait if $y_{\mathrm{ik}}=0$	
First item	Second item	First item	Second item	First item	Second item
+	+	1	0	0	1
+	−	1	1	0	0
−	+	0	0	1	1
−	−	0	1	1	0

*Note.* If the first item is preferred over the second, this comparison is coded with 1. If the second item is preferred over the first, this comparison is coded with 0.

This scoring scheme closely resembles the scoring in Thurstonian IRT models with factor loadings constrained to 1 in disregard of the intertrait correlation matrix. The scheme reveals that equally and unequally keyed items contain two different types of information. While responses to equally keyed items provide information about the relative standing of traits within one person (but not about the absolute scores), unequally keyed items contain information about absolute trait location compared with other persons (assuming trait directions to be aligned; otherwise the role of equally and unequally keyed items switches). However, one cannot state which of the two latent traits within the paired comparison has the higher value based on unequally keyed items. One consequence of this is that scores based on mixed keyed items are no longer fully ipsative in the sense that the sum of trait scores are no longer constant between individuals. Thus, this effect is not an exclusive property of T-IRT but rather a general characteristic of FC questionnaires that contain mixed keyed items.

For all T-IRT models we used the *thurstonianIRT* package (Bürkner, 2019) in R (R Core Team, 2018) with Stan as the underlying engine. Stan is a programming language for Bayesian statistics (Carpenter et al., 2017). The *thurstonianIRT* package uses expected a posteriori (EAP) estimation and weakly informative default priors. Priors largely avoid convergence issues frequently observed in frequentist implementations of T-IRT (Bürkner et al., 2019). The priors do not influence parameter estimation considerably, and the Stan-based estimates correlated perfectly ( r≥.98 ) with the frequentist implementation in Mplus (Muthén & Muthén, 2015) introduced by Brown and Maydeu-Olivares (2012) in previous simulations (see Table 6 in Bürkner et al., 2019). Item and person parameters were estimated jointly in each model. The R code for all simulations and the postprocessing are available on OSF (https://osf.io/t6krs/).

### Measures of Parameter Recovery

The measurement precision of person parameter estimates was operationalized as (a) the reliability and (b) the RMSE. Reliability is defined as the proportion of true variation relative to the total variation in the person parameters:

(4) $$\mathrm{Rel}(\hat{\theta },\theta )=\mathrm{Cor}(\hat{\theta },\theta {)}^{2}.$$

In Equation (4), θ represents the true and $\hat{\theta }$ the estimated person parameters. As a measure of absolute fit, we will investigate the RMSE on a z -scale:

(5) $$\mathrm{RMSE}(\hat{\theta },\theta )=\sqrt{\frac{1}{N}\sum_{i=1}^{N}{\left({\hat{\theta }}_{i}-\theta_{i}\right)}^{2}},$$

with N being the number of persons over which the RMSE is computed.

We evaluated the bias in the estimation of intertrait correlations by subtracting the true intercorrelations from their estimates. The intercorrelational bias not only indicates how well the true covariance structure is recovered but can also be an indication that model estimates suggest scale interdependencies where they actually do not exist. Another more direct way to look at ipsativity issues is that they prevent the identification of individuals who score low/high on all of the measured scales, that is, person mean differences cannot be recovered. Therefore, we calculated the correlations between individuals’ true and estimated score means over all measured traits:

(6) $$\mathrm{Cor}(\hat{\bar{\theta }},\bar{\theta })$$

with $\hat{\bar{\theta }}$ being the mean of the estimated person parameters $\theta_{i}$ of person i over all measured traits and $\bar{\theta }$ being the mean of the true person parameters $\theta_{i}$ of person i over all measured traits. If the variance of person means within one condition was zero, the corresponding correlations were not defined. This applies in particular to the standard scorings, where person means have no variance between persons as scores are perfectly ipsative. For illustrational purposes, we display these correlations as zero in the graphics depicting the simulation results (with a note that they are not actually zero but undefined).

To quantify the uncertainty of model accuracy indicators, we calculated approximate confidence intervals based on their variance between the k trials of the same simulation condition. For instance, the uncertainty of reliability estimates was quantified as follows:

(7) $$\bar{\mathrm{Rel}}\pm 1.96\times \frac{SD_{\mathrm{Rel}}}{\sqrt{k}}$$

with $\bar{\mathrm{Rel}}$ being the mean and $SD_{\mathrm{Rel}}$ being the standard deviation of all k reliability estimates in this condition. Approximate confidence intervals for other indicators were calculated analogously.

## Results

**Figure 1.**
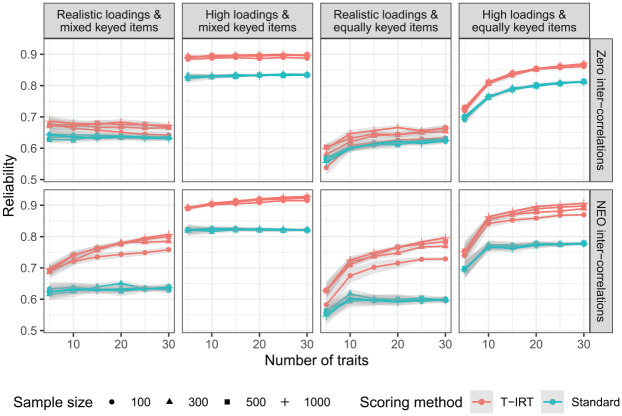
Reliability estimates. Realistic loadings = factor loadings from N(.5,.16) , high loadings = uniform(.65,.95) , NEO intercorrelations are taken from the German NEO-PI-R. Gray shaded areas are approximate confidence intervals.

Figure 1 shows the reliability estimates for all simulated conditions. Overall, the factor loadings exhibit the strongest effect. Higher loadings lead to more precise estimates in both T-IRT and classical scorings. For scales with high factor loadings, T-IRT scores (red lines) reach reliabilities of .8 or higher if items are mixed (i.e., half negatively) keyed or if items are equally keyed and 10 or more traits are measured. In contrast, when more realistic factor loadings are used, the reliability is considerably lower. The sample size has little effect. Only when N=100 does the reliability drop in some conditions. As expected, T-IRT performs better than classical scores (blue lines) in most conditions, but the difference does not exceed .1 units on the reliability scale. The number of traits shows differential effects depending primarily on factor loadings (size and sign) and intercorrelation. In some conditions, it shows no effect within the range of five to 30 traits. In other conditions (e.g., zero intercorrelations, high loadings & equally keyed items), the highest increase in reliability is observed between five and 10 traits followed by an accumulating saturation. For conditions where the reliability is constant within the range of five and 30 traits (e.g., for high loadings, mixed keyed items and zero intercorrelations), it can be assumed that this saturation occurs already within the range of fewer than five traits. The intercorrelations have no influence on the classical scores but do influence T-IRT scores. With T-IRT, more reliable estimates are obtained if traits are intercorrelated in a realistic manner.

**Figure 2.**
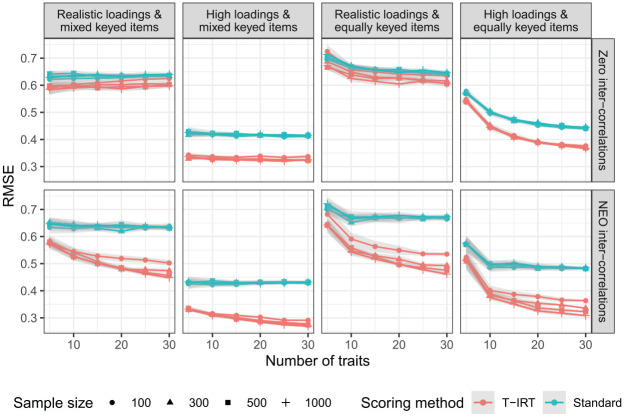
Root mean square error (RMSE) estimates for all simulation conditions. Realistic loadings = factor loadings from N(.5,.16) , high loadings = uniform(.65,.95) , NEO intercorrelations are taken from the German NEO-PI-R. Gray shaded areas are approximate confidence intervals.

Figure 2 shows the results for the RMSE as a second indicator of measurement accuracy. The RMSE describes the absolute measurement error and gives direct information about the expected accuracy of an individual measurement in the units of the measured scale. The patterns between conditions mirror those of the reliabilities. The absolute values are high with respect to most real-world questions. For the practical relevant conditions (realistic loadings and intercorrelations, equally keyed loadings), the T-IRT-based RMSE does not fall below .46 even under the most favorable circumstances ( N=1000 , $N_{\mathrm{traits}}=30$ ). Under the assumption of normally distributed person parameter estimates, this yields a confidence interval of [−1.96×.46,1.96×.46]=[−.90,.90] on a z -scale for an average person, covering 63% of the population. Compared with the *RMSE*s of the T-IRT estimates, those based on the standard scoring are equal or higher. For the same condition as described before, the standard scoring yields an *RMSE* of .67 , and therefore, results in an even larger confidence interval of [−1.31,1.31] .

**Figure 3.**
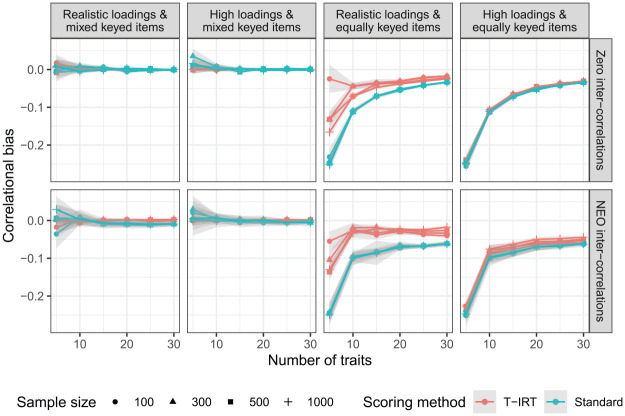
Bias of intertrait correlations. Realistic loadings = factor loadings from N(.5,.16) , high loadings = uniform(.65,.95) , NEO intercorrelations are taken from the German NEO-PI-R. Gray shaded areas are approximate confidence intervals.

Next, we turn our attention to the estimates of intertrait correlations (Figure 3). When mixed keyed items are used, the estimates are very accurate for both T-IRT and the standard scoring procedure. Only for five traits does the accuracy seems to be imperfect but still satisfactory. For equally keyed items, we see a clear bias in the estimation of intertrait correlations. The pattern of results is similar for T-IRT and standard scores. It decreases drastically between five and 10 traits and is around .1 or smaller if 10 or more traits are measured. For classical scores, the pattern does not differ by factor loading or intercorrelation. T-IRT models do not benefit from the incorporation of factor loadings. In the condition of high factor loadings, T-IRT does not recover the covariance structure better than the classical scoring approach, neither for zero intercorrelations nor for those from the NEO-PI-R. The sample size does not influence the bias considerably.

**Figure 4.**
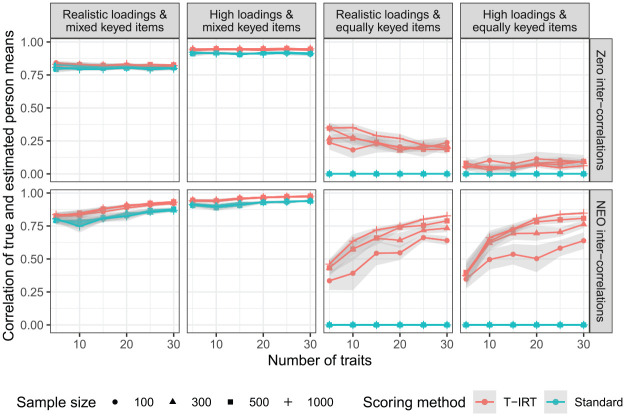
Correlation of true and estimated person means. Realistic loadings = factor loadings from N(.5,.16) , high loadings = uniform(.65,.95) , NEO intercorrelations are taken from the German NEO-PI-R. Gray shaded areas are approximate confidence intervals.

Finally, Figure 4 shows the correlation of true and estimated person mean scores for all conditions. Both scoring methods yield good results for mixed keyed items. This is true for the whole range of sample sizes and trait numbers considered here. Based on the correlation of true and estimated person means, the person parameter estimates seem to have a normative quality. A perfect correspondence between true scores and estimates is not reached, but this could be expected due to the unreliability of the trait estimates. In contrast, for equally keyed items, the means of true and estimated parameters based on the standard scoring do not correlate. To be precise, the correlation is undefined due to the zero variance, and we set it to zero for illustrational purposes demonstrating the ipsative nature of these measures. For T-IRT estimates, the correlations are also low when traits are not intercorrelated. In the respective conditions, the increase of trait numbers does not exhibit any positive effect. In contrast, under realistic intercorrelations, correlations between true person means and T-IRT person means are higher and increase as the number of measured traits goes up. Yet the absolute levels remain below those achievable with mixed keyed items. Even under optimal conditions (high loadings and 30 traits), the correlation does not exceed .85. In comparison, the conditions with mixed keyed items show that the variation in the estimated person means of equally keyed items that is not explained by the true person means cannot exclusively be attributed to the unreliability of trait scores. Instead, the low correlation of true and estimated person means for equally keyed items is an indicator of partial ipsativity. The ipsativity tendency seems to be more pronounced in very small sample sizes (i.e., if N=100 ).

In our simulations, the factor loadings (i.e., their size, not their sign) were randomly drawn and randomly combined into blocks. A reviewer recommended to improve reliability by optimizing the combination of items by maximizing squared difference of factor loadings within each block. We tried this on a sample of our simulation conditions and could not observe any improvement. To ensure that higher numbers of trials would not change the results, we also computed the most relevant conditions (realistic factor loadings, realistic inter-correlations, equally-keyed items) again with 120 instead of 12 trials per condition. The results did not change notably due to the increase of the trial number. The analysis scripts and results for both additional analyses can be found in the OSF (https://osf.io/t6krs/)

## Discussion

The aim of this study was to determine the minimal number of traits necessary to obtain reliable and nonipsative (i.e., normative) T-IRT person parameter estimates. Additionally, we compared T-IRT scores with those from the classical scoring method and investigated the influence of the sample size and factor loadings on the estimates.

### Properties of T-IRT Scores

Results show that the excellent reliabilities for 30 traits observed in Bürkner et al. (2019) are limited to questionnaires with high factor loadings used in those simulations. If factor loadings are more realistic, reliability estimates are less than .80, and confidence intervals for real-world test scores cover wide parts of the population. For tests with equally keyed items, the reliability drops drastically, even in conditions with high factor loadings if less than 10 traits are measured.

Further, our results indicate that estimates remain partially ipsative when equally keyed items are used. The negative bias of intercorrelations observed in our simulations is a typical property of ipsative questionnaires (Guilford, 1952; Hicks, 1970). The low correlations of true and estimated person means when items are equally keyed compared with conditions with unequally keyed items also point to the ipsative nature of these scores. Normative information in the factor scores seems to originate primarily from the incorporation of trait intercorrelations into the model, and to a far lesser extent, from the free variation of factor loadings. If person parameters remain partially ipsative, this raises doubts about many of the validity estimates reported for T-IRT. This is because the sum of the covariance terms obtained between a specified criterion and a set of fully ipsative variables is zero (Clemans, 1966; Hicks, 1970). Consequently, fully ipsative measures cannot be interpreted regularly and compared with validity estimates from normative measures. T-IRT estimates are not completely ipsative, but their correlation with external criteria might still be biased due to their partially ipsative nature. The published validity estimates for T-IRT estimates are typically comparable with rating scales (Anguiano-Carrasco et al., 2015; Brown & Maydeu-Olivares, 2013; Lee et al., 2018; Watrin et al., 2019; Wetzel & Frick, 2019). However, when effect sizes in favor of one method are small, even limited bias can change the preference of one method over the other.

The sample size had little effect on the properties of T-IRT models, at least within the simulated range. Only in some conditions did model properties improve slightly between N=100 and N=300 . Thus, the sample size at which additional persons do not improve model estimates lies between 100 and 300. Any further increase was without noteworthy effect.

### Comparison Between T-IRT and Traditional Scoring

Comparing T-IRT with classical scoring procedures, T-IRT yields equal or better results. The measurement precision is typically higher, and under realistic conditions simulated here, the intercorrelational bias is lower. Most important, T-IRT allows for less ipsative person parameter estimates when items are equally keyed. However, if the number of measured traits is low (e.g., 5 in our simulations), the *RMSE* of both methods is comparable, which helps explain the set of validation studies that do not favor T-IRT when compared with classical scorings (Fisher et al., 2019; Lee et al., 2018; Walton et al., 2019; Wetzel, Roberts, et al., 2016).

In the past, some researchers have claimed that T-IRT solves the problem of ipsativity and suggested the use of mixed keyed items at the same time. Against the background of our results, this seems implausible. Not only do mixed keyed items serve as a backdoor that reintroduces faking options into the FC format but also, when factor loadings are mixed (i.e., half negatively) keyed, the intercorrelational bias is similar (low loadings) or equal (high loadings) for T-IRT and classical scores. Besides that, true and T-IRT-based person means correlate much more weakly for equally keyed items than for unequally keyed ones, pointing out the (partially) ipsative nature of these scores. Still, practitioners for whom the much more complicated T-IRT method is not a hurdle might benefit from the moderate but consistent reliability increase of T-IRT as compared with classical scorings when a high number of traits is measured.

In comparison with earlier simulations on the standard scoring technique (Bartram, 1996; Saville & Willson, 1991), we found similar or lower reliabilities. One study found a slightly higher reliability of .92 for 32 traits, and these dropped on a still remarkable level of .79 for seven uncorrelated traits (squared values from Table 3 in Saville & Willson, 1991). Definite conclusions about the reasons for the disparate results cannot be drawn, as conditions do not match on more than one variable and the data generation processes differ drastically between the studies. Nonetheless, we assume the main reasons to be the very high correlations of .9 among items measuring the same trait or the uniform distribution of true scores used by Saville and Willson (1991), or a combination of both.

In a second simulation (Bartram, 1996), normally distributed normative scores were correlated with ipsative scores derived from normative scores by subtracting the mean for each person over all scales for each of the scale scores. Results suggested that normative and ipsative scores correlate highly when scales are independent of each other ( r=.96 for 12 traits and .98 for 30 traits). For moderate correlations of .3 and high numbers of traits, the correlation was still considerable ( r=.81 for 12 traits and .82 for 30 traits). Further, the authors introduced a formula which derives reliabilities of ipsative scales from the reliability of their normative counterparts (Bartram, 1996, Equation 10). They conclude that under some circumstances, ipsatized scales can be reasonably reliable. Based on our simulations, this conclusion can be generalized to our method of ipsatization, that is, direct ipsative measures derived from FC questionnaires. However, our results show that under the realistic conditions simulated here – especially when factor loadings drop to a typical level – the measurement precision of the standard scoring method falls off notably and does not meet accepted standards.

### Practical Implications

Our results raise doubts about whether high psychometric standards can be met under realistic conditions with T-IRT. When factor loadings are not extraordinarily high, reliability estimates are below .80 and, therefore, are too low for individual diagnostic purposes in high-stakes situations. Test developers still considering the use of the technique should note that the estimate quality drops off notably if few (i.e., five) traits are measured.

Some test developers might consider the development of high-dimensional questionnaires with items showing very high factor loadings. To reach the necessary levels of factor loadings, the measured constructs would have to be extremely homogeneous, allowing only for the prediction of very specific criteria. In contrast, the predictive validity for broad constructs would be limited. Besides that, the use of questionnaires with an excessive number of scales for personnel selection has been criticized, as they are unlikely to be empirically independent and do not necessarily predict unique, meaningful proportions of employee job performance (Meade, 2004).

Empirically, most personality questionnaires form a factor structure of about five distinct dimensions (Goldberg, 1993). The five-factor model, however, comprises personality facets that are too heterogeneous to be covered by items with extremely high loadings. Such high loadings can only be achieved by extremely similar items, but these are inadequate as content-valid operationalizations of broad constructs. What remains as a strategy for test development is the use of mixed keyed items. From a practical point of view, it seems to be a paradox to develop an FC questionnaire with item pairs that might reintroduce the possibility of faking, which is likely the case when one item (i.e., the one with the negative factor loading) represents the undesired end of the trait continuum.

In contrast, the measurement precision of T-IRT scores based on equally keyed items with realistic factor loadings and intercorrelations is weak. Even when a very high number of traits is measured, the confidence intervals of individuals’ trait scores are very wide. Given that in applied contexts the reliability is further diminished by additional factors like fatigue and distraction of respondents, we would refrain from the development of such a questionnaire.

### Limitations

Despite our extensive simulations, some limitations need to be mentioned. First, some factors influencing the model accuracy were not varied but held constant. In particular, this affected the block size (three items per block), the response format (RANK), and the number of blocks per trait (nine in these simulations). However, higher block sizes question the appropriateness of the theoretical assumption that responses can be described by the *Law of Comparative Judgment* (Thurstone, 1927) adequately. The RANK format is the optimal response format for FC items because it generates the highest amount of information (Hontangas et al., 2015). The number of blocks per trait has a small but relevant influence on the precision of estimates (Bürkner et al., 2019), but for high-dimensional questionnaires this number cannot be increased arbitrarily due to time limitations. This study was designed to determine the best performance of T-IRT models that is possible under realistic conditions. Therefore, we did not apply a condition with exclusively positive intercorrelations (Bürkner et al., 2019) and lower proportions of mixed keyed items, because it can be assumed that this would lead to even lower measurement precisions.

Second, there are other item configurations that we have not examined. These include unidimensional comparisons and the insertion of distractor items. It cannot be ruled out that these will lead to improvements, as they may contain more information on absolute trait standing.

Third, we assumed that high scores on all measured traits are ideal. In practice, there are cases where some traits are considered more important than others (e.g., in a selection scenario) or medium trait level (e.g., for agreeableness) are desirable. Yet we argue that when test-takers can identify less attractive traits, any FC technique is pointless because highly attractive and less attractive items will be compared. Furthermore, scales for which medium values are desired are not optimally modelled by the Thurstonian IRT models. If we understand the utility of an item in a faking-prone context as a function of a person’s true value and the item’s social desirability, and medium scores are desirable, the utility will have a peak in the middle of the latent scale. T-IRT models are based on the dominance approach that assumes true trait values and latent utilities to be linearly related, and therefore, cannot model such situations adequately. Ideal point models (Coombs, 1960; Stark et al., 2005) might be more appropriate in such a scenario.

Fourth, our results are based on 12 trials per simulation condition. The reason for this comparably low number is that the model estimation in our simulation is computationally very intensive and requires several days per model for the larger models. However, the low dispersion within conditions led us to assume that more trials would not have changed the results substantially (Bürkner et al., 2019). In the conditions for which we computed 120 trials, we also did not see any relevant differences between the results of 12 and 120 trials (see supplementary material on https://osf.io/zk79h/).

### Future Research

Based on our results, it seems to be unlikely to derive precise and normative person parameter estimates from a faking-resistant FC test using either classical or T-IRT methods to score responses. However, other scoring approaches have been proposed (for an overview, see Brown, 2016). Even though there is a certain likelihood that all scoring methods suffer from the limited information FC responses provide on the absolute trait standing, better performance of other models should not be ruled out. One alternative in this context is the multi-unidimensional pairwise preference model (Stark et al., 2005), which is based on an ideal point approach (Coombs, 1960). On the one hand, among the available alternatives this model has comparatively better chances to lead to improvements, because it is less similar to the T-IRT model than others. However, the ideal point parameter might play a similar problematic role for the identification of the scale origin as the factor loadings in T-IRT models (Brown, 2016, Equation 44) and probably cannot be disregarded with respect to matching for social desirability either.

Another way to improve the measurement precision of FC questions are graded paired comparisons. This format uses a rating scale between two items. While this drastically increases the information generated with each item, it also allows for response biases such as extremity and midpoint tendency. Regarding its faking resistance, this format is promising, because it maintains the mechanism that not all attractive items can be fully endorsed. An ordinal scoring method for this kind of questionnaire data has been introduced recently (Brown & Maydeu-Olivares, 2018).

An alternative scoring approach should be validated appropriately. The minimum requirements for a convincing validation study would be (a) a questionnaire that is faking resistant what should at least be ensured by employing a reasonable matching method that allows for the desirability of items within each block to be equal. Such a questionnaire would have to correlate (b) with a relevant criterion not measured with FC or rating scales and (c) the validity estimates should be higher for T-IRT FC scales than for the rating version of that questionnaire and (d) other FC scoring methods in (e) a high-stakes situation. Until now, no such validation attempt has been published.

### Conclusion

Taken together, our results show that T-IRT models represent significant progress in the scoring of responses to FC tests. They perform equally well or better than the traditional scoring technique. This is not surprising, given that traditional scoring methods do not account for varying factor loadings and intertrait correlations. While the improvements in measurement properties are often gradual, the most remarkable enhancement relates to the ipsativity of scores from equally keyed blocks measuring intercorrelated traits. Under these conditions, classical scores are completely ipsative, whereas T-IRT scores incorporate a considerable amount of nonipsative variance, especially if the number of measured traits is high.

Nonetheless, for excellent measurement precision as is required in high-stakes situations for decisions on the individual level, T-IRT models can only be applied for questionnaires with very high factor loadings and mixed keyed items within blocks. As FC tests are hardly faking resistant if mixed keyed items are used, this condition is not consistent with the motivation that led to the development and application of FC questionnaires in the first place. Another approach to construct a T-IRT analyzed FC questionnaire would be to measure 10 or more dimensions using items with very high factor loadings. In practice, this is not the typical condition under which psychometric tests can be developed. Additionally, our results suggest that scores remain partially ipsative in this scenario.
